# Moving Object Detection Using Dynamic Motion Modelling from UAV Aerial Images

**DOI:** 10.1155/2014/890619

**Published:** 2014-04-29

**Authors:** A. F. M. Saifuddin Saif, Anton Satria Prabuwono, Zainal Rasyid Mahayuddin

**Affiliations:** ^1^Faculty of Information Science and Technology, Universiti Kebangsaan Malaysia (UKM), 43600 Bangi, Selangor Darul Ehsan, Malaysia; ^2^Faculty of Computing and Information Technology, King Abdulaziz University, P.O. Box 344, Rabigh 21911, Saudi Arabia

## Abstract

Motion analysis based moving object detection from UAV aerial image is still an unsolved issue due to inconsideration of proper motion estimation. Existing moving object detection approaches from UAV aerial images did not deal with motion based pixel intensity measurement to detect moving object robustly. Besides current research on moving object detection from UAV aerial images mostly depends on either frame difference or segmentation approach separately. There are two main purposes for this research: firstly to develop a new motion model called DMM (dynamic motion model) and secondly to apply the proposed segmentation approach SUED (segmentation using edge based dilation) using frame difference embedded together with DMM model. The proposed DMM model provides effective search windows based on the highest pixel intensity to segment only specific area for moving object rather than searching the whole area of the frame using SUED. At each stage of the proposed scheme, experimental fusion of the DMM and SUED produces extracted moving objects faithfully. Experimental result reveals that the proposed DMM and SUED have successfully demonstrated the validity of the proposed methodology.

## 1. Introduction

Moving object extraction observed in a video sequence and estimation of corresponding motion trajectories for each frame are one of the typical problems of interest in computer vision. However, in real environments moving object extraction becomes challenging due to unconstraint factors that is, rural or clutter environment, brightness or illumination, static or dynamic object types which together motion degeneracy may result in worthless for moving object extraction [1–35]. Besides, due to rapid platform motion, image instability, the relatively small size of the object of interest signatures within the resulting imagery, depending on the flight altitude and camera orientation, appearance of the objects within the observed environment changes dramatically making the moving object detection a challenging task [27–31, 36–60].

Motion carries a lot of information about moving object pixels which plays important role for moving object detection as image descriptor to provide a rich description of object in different environment [11, 61–65]. From physics perspective, it found its way into probability theory, and since computer vision leverages probability theory, this research finds that it is only natural to use moments to develop a dynamic motion modeling which can be applied or becomes helpful to limit segmentation tasks in computer vision to reduce computational complexity.

This paper presents moments based motion parameter estimation called dynamic motion model (DMM) to limit the scope of segmentation called SUED which influences overall detection performance. Based on analysis from previous moving object detection frameworks, this paper used DMM model which is embedded under frame difference based segmentation approach and can handle robustness for optimum detection performance.

## 2. Background Study

For accurate detection, motion must be accurately detected using suitable methods which are affected by a number of practical problems such as motion change over time and unfixed direction of moving object. Motion pattern analysis before detecting each moving object has started to get attention in recent years, especially for crowded scenarios when detecting each individual is very difficult [46]. Through the modelling of object motion, the detection task becomes easy and thus also can handle noise.

**Figure 1:**
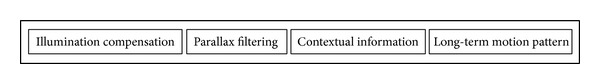
Existing approaches for motion based object detection.

As the scene may contain different motion patterns at one location within a period of time, that is, road intersection, averaging or filtering before knowing the local structure of motion patterns may destroy such structure. This paper proposes to use effective motion analysis for moving object extraction. In Figure 1, four existing approaches for motion analysis are shown.

Global illumination compensation approach works on brightness or illumination changes. Due to dependency on brightness, in real world this research does not progress so far. In parallax filtering approach, a scene that contains strong parallax is still difficult for existing methods to achieve good segmentation results [46]. In contextual information approach, contextual information has been applied to improve detection of moving object from UAV aerial images [46, 48]. This method assumes low level motion detection. The errors in low level motion segmentation under strong parallax situation are not considered in this method. For long-term motion pattern analysis [46] approach, there is scope to use context information to improve both low level motion segmentation and high level reacquisition even when there is only one single object in the scene [46]. However, this approach still needs further research to detect moving object robustly.

Table 1 shows the comparison table for compatibility of different approach for motion based moving object detection research from UAV aerial images. In Table 1, low level of motion indicates motion without parallax; high level motion indicates motion with parallax. Among these 4 types, the first three are not distinctive property for moving object because of environmental condition for the first types; a lot of parameter calculations for the second, third, and fourth types increase computation complexity.

Indeed, detection of motion and detection of object are coupled. If proper motion detection is done, detection of moving object from UAV aerial image becomes easier. Very few researches concentrate on adaptive robust handling of noise and unfixed motion change as well as unfixed moving object direction. For that reason an adaptive and dynamic motion analysis framework is needed for better detection of moving object from UAV aerial images where overall motion analysis reduces dependency on parameter. In other words, detection of motion indicates detection of motion pixels from frames which can be described as some function of the image pixel intensity. Pixel intensity is nothing but the pixel color value. Moments are described with respect to their power as in raised-to-the-power in mathematics. Very few previous researches used image moments to present motion analysis. Thus, this paper proposes to use image moments before segmenting individual objects and to use motion pattern in turn to facilitate the detection in each frame.

Moving object detection from UAV aerial images involves dealing with proper motion analysis. Previously very few researchers used methods which involve effective motion analysis. In [1, 16], the authors proposed Bayesian network method which depends on the fixed shape object constraint and also did not overcome the problem of aspect ratio constraint. Image registration method does not suit because as the number of motion block increases the detection rate decreases. Clustering based approach does not suit well for the complexity of shortening environment and inconspicuous features of object [10, 17]. Scalar invariant feature transform (SIFT) used only key point of object and does not suit well for noisy environment [6]. Cascade classifier based approach uses gray scale input images which are unrealistic in the real-time object detection [9]. Symmetric property based approach gave good result only on structural object shape [5, 11]. Background subtraction approach does not overcome blob-related problems mentioned above for registration method [8, 13, 38]. Shadow based approach depends on lightening condition [2, 7, 59]. Region based appearance matching approach does not give the optimum result for crowded scenarios [44, 54]. Histogram oriented gradients (HOG) approach rejects objects backgrounds [36].

**Figure 2:**
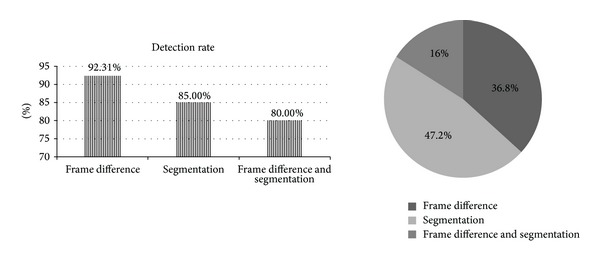
Detection rate [1, 15, 36, 40] and percentage of using frame difference and segmentation approach in the previous research.

Among these methods only frame difference approach is involved with motion analysis although most of previous research does not provide proper motion estimation to handle six uncertainty constraint factors (UCF) [29]. Frame difference causes the moving object to become into pieces due to object's color homogeneity and also grabs the motion information. Frame difference based approaches were performed by registering two consecutive frames first, followed by frame difference to find moving objects. These approaches are faster but usually cause a moving object to become into pieces especially when the object's color distribution is homogenous. Frame difference detects the pixels with motion but cannot obtain a complete object [14, 37]. Using segmentation based approach can overcome the shortcomings of using frame difference based approach. Image segmentation is used to determine the candidates of moving objects in each video frame [42]. Image segmentation extracts a more complete shape of the objects and reduces computation cost for moving object detection from UAV aerial images [39, 47]. However, it does not have the ability to distinguish moving regions from the static background. Using frame difference and segmentation together can achieve optimum detection performance but research in [42, 44] did not achieve reliable performance because of approximating pure plane and complexity needs to be low. Low detection rate and high computation time are the current research problems to apply frame difference and segmentation together [1, 15, 36, 40] as shown in Figure 2. Applying frame difference or segmentation separately does not include motion analysis currently which increases detection rate with low computation complexity.

This paper states that as frame difference cannot obtain motion for the complete object alone and segmentation does not have the ability to differentiate moving regions from the basic static region background, so applying frame difference and segmentation together is expected to give optimum detection result with high detection speed for moving object detection from UAV aerial images instead of applying frame difference or segmentation separately. For that reason this paper proposes moments based motion analysis to apply under frame difference based segmentation approach (SUED) which ensures robustness of the proposed methodology.

## 3. Research Methodology

Proposed moments based motion modeling is depicted in Section 3.1 and frame difference based segmentation approach, segmentation using edge based dilation, is depicted in Section 3.2. Each section of methodology is proposed with new approach to ensure robustness and accuracy of detection approach.

### 3.1. Proposed Dynamic Motion Model (DMM)

**Figure 3:**
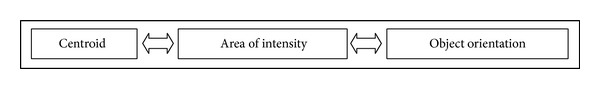
Properties of image moments.

In computer vision information theory, moments are the uncertainty measurement of the object pixels. Besides, an image moment is a certain particular weighted average (moment) of the image pixel's intensities, or a function of such moments, usually chosen to have some attractive property or interpretation. Image moments are useful to describe objects after segmentation. Properties of the image which are found via image moments are centroid, area of intensity, and object orientation as shown in Figure 3.

Each of these properties needs to be invariant by the following terms: translational invariant, scale invariant, and finally rotation invariant. Any feature point is called translational invariant if it does not distinguish under different points in space. Or translational invariant means that a particular translation does not change the object. Scale invariance is a feature of objects or laws that does not change if scales of length, energy, or other variables are multiplied by a common factor. The technical term for this process is known as dilation. A feature point is said to have rotation invariant if its value does not change when arbitrary rotations are applied to its argument.

**Figure 4:**
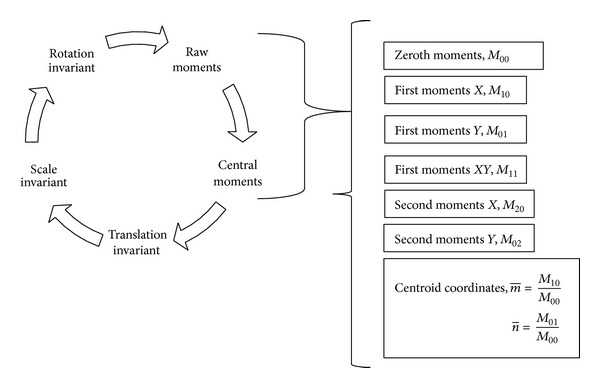
Flow of moments calculation in the proposed research.

Image moments can simply be described as some function of the image pixel intensity. Pixel intensity is nothing but the pixel color value. Moments are described with respect to their power as in raised-to-the-power in mathematics. This research calculates zeroth moment, first moment, second moment, and so forth from raw moments. Later this research transformed these moments into translational, scale, and rotation invariant. This research presents the following organized structure to calculate moments shown in Figure 4.

#### 3.1.1. Raw Moments

Before finding central moments it is necessary to find raw moments of *FD*(*m*, *n*) in *t* frame video sequence. If $\overset{-}{m}$ and $\overset{-}{n}$ are the components of the centroids, raw moments of *FD*(*m*, *n*) for (*p* + *q*) can be defined as

(1) $$\begin{array}{c} M_{pq}=\sum_{p}\sum_{q}m^{p}n^{q}FD\left(m,n\right). \end{array}$$

In case of considering *FD*(*m*, *n*) as a 2D continuous function, (1) can be expressed as

(2) $$\begin{array}{c} M_{pq}=\iint m^{p}n^{q}FD\left(m,n\right), \end{array}$$

where centroid coordinates are as follows:

(3) m−=M10M00,  n−=M01M00,M00=zeroth  moment=∑m∑nm0n0FD(m,n)=∑m∑nFD(m,n),M10=first  moment  X=∑m∑nm1n0FD(m,n)=∑m∑nmFD(m,n),M01=first  moment  Y=∑m∑nm0n1FD(m,n)=∑m∑nnFD(m,n),M11=first  moment  XY=∑m∑nm1n1FD(m,n)=∑m∑nmnFD(m,n),M20=second  moment  X=∑m∑nm2n0FD(m,n)=∑m∑nm2FD(m,n),M02=second  moment  Y=∑m∑nm0n2FD(m,n)=∑m∑nn2FD(m,n).

#### 3.1.2. Central Moments

Using centroid coordinates, central moments for *FD*(*m*, *n*) can be defined as

(4) $$\begin{array}{c} \mu_{pq}=\sum_{p}\sum_{q}{\left(m-\overset{-}{m}\right)}^{p}{\left(n-\overset{-}{n}\right)}^{q}FD_{d}\left(m,n\right). \end{array}$$

*(1) Translation Invariant.* To make *FD*_*d*_(*m*, *n*) translation invariant, *μ*_*pq*_ can be defined as

(5) (i) μpq=∬(m−m−)p(n−n−)qFDd(m,n)dm dn, (ii)  μpq=∬∑r=0p(pr)×mr·(−m−)(p−r)×∑s=0q(qs)ns·(−n−)(q−s)FDd(m,n)dm dn(by  using  the  formula:(a+b)k=∑r=0k(kr)ar·b(k−r)), (iii)  μpq=∑r=0p∑s=0q(pr)(qs)(−m−)(p−r)(−n−)(q−s)    ×∬×mr·×ns·FDd(m,n)dm dn[rearranging], (iv)  μpq=∑r=0p∑s=0q(pr)(qs)(−m−)(p−r)(−n−)(q−s)·Mpq[using  (2)].

Equation (5) is central moments of *FD*_*d*_(*m*, *n*) which is translation invariant and derived from raw moments.

*(2) Scale Invariant. *Let *FD*′(*m*, *n*) be the scaled image of *FD*(*m*, *n*) scaled by *λ*. So,

(6) $$\begin{array}{c} {FD}^{\prime }\left(m,n\right)={FD}^{\prime }\left(\frac{m}{\lambda },\frac{n}{\lambda }\right), \end{array}$$

where

(7) $$\begin{array}{c} m^{\prime }=\frac{m}{\lambda },\;m=\lambda m^{\prime },\;dm=\lambda dm^{\prime },\\ n^{\prime }=\frac{n}{\lambda },\;n=\lambda n^{\prime },\;dn=\lambda dn^{\prime }. \end{array}$$

From (2), the following equation can be written:

(8)  (i)  μpq′=∬mpnqFD(mλ,nλ)dm dn, (ii)  ∬(λm)p(λn)qFD(m′,n′)·λdm′·λdn′, (iii)  μpq′=λpλqλ2∬(m)p(n)qFD(mλ,nλ)dm′dn′, (iv)  μpq′=λp+q+2μpq [Using  (2)].

For *p* = 0; *q* = 0 and assuming total area to be 1, (8) becomes

(9)  (i)  μ00′=λ2μ00, (ii)  λ2μ00=1, (iii)  λ2=1μ00, (iv)  λ=μ00−(1/2).

Putting *λ* into (8) becomes *η*_*pq*_ = (*μ*_00_)^−(1/2)·*p*+*q*+2^ · *μ*_*pq*_,

(10) $$\begin{array}{c} \text{(i) }\eta_{pq}=\frac{1}{{\left(\mu_{00}\right)}^{\left(p+q+2\right)/2}}\cdot \mu_{pq}. \end{array}$$

Equation (10) is scale invariant central moments of *FD*(*m*, *n*).

*(3) Rotation Invariant. *Let *FD*′(*m*, *n*) be the new image of *FD*(*m*, *n*) rotated by *θ*. So,

(11) $$\begin{array}{c} {FD}^{\prime }\left(m,n\right)=f\left(m\cos\theta +n\sin\theta ,-m\sin\theta +n\cos\theta \right). \end{array}$$

Using variable transformation,

(12) $$\begin{array}{c} m^{\prime }=m\cos\theta +n\sin\theta ; \end{array}$$

(13) $$\begin{array}{c} m=m^{\prime }\cos\theta -n^{\prime }\sin\theta ; \end{array}$$

(14) $$\begin{array}{c} dm=\cos\theta dm^{\prime }, \end{array}$$

(15) $$\begin{array}{c} n^{\prime }=m\sin\theta +n\cos\theta , \end{array}$$

(16) $$\begin{array}{c} n=m^{\prime }\sin\theta +n^{\prime }\cos\theta , \end{array}$$

(17) $$\begin{array}{c} dn=\cos\theta dn^{\prime }. \end{array}$$

Using (2),

(18)  (i)  μpq′=∬mpnqFDd′(m,n)dm dn, (ii)  μpq′=∬mpnqf′×(mcos⁡θ+nsinθ,−msinθ+ncos⁡θ)dm dn[Using  (11)],(iii)  μpq′=∬(m′cos⁡θ−n′sinθ)p(m′sinθ+n′cos⁡θ)q×FDd(m′,n′)cos⁡2θdm′dn′[Using  (11)–(17)].

So rotation invariant *θ* can be expressed as

(19) $$\begin{array}{c} \theta =\frac{1}{2}\arctan\left(\frac{2\mu_{11}}{\mu_{20}-\mu_{02}}\right). \end{array}$$

Here *θ* or (19) is the rotation invariant central moment measurement of *FD*(*m*, *n*).

**Figure 5:**
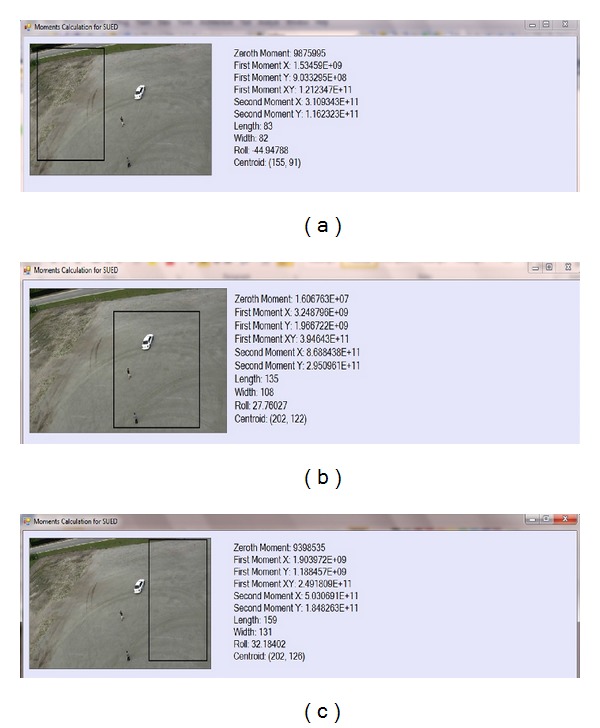
(a) Search window 1 for *FD*(*m*, *n*) in *t* time. (b) Search window 2 for *FD*(*m*, *n*) in *t* time. (c) Search window 3 for *FD*(*m*, *n*) in *t* time.

Output of calculated moments proposed in this research is depicted in Figures 5(a)–5(c). In the search window, the higher the moments value, that is, first moments *XY*, more higher probability that search window contains moving object.

Before going to newly proposed SUED algorithm using DMM model, input frame needs to be projected on the search (black box) only to reduce the risk of extraction failure. This research emphasizes that using a search window around the original object is very important. It limits the scope of segmentation to a smaller area. This means that the probability of the extraction is getting lost because a similar coloured object in the background is reduced. Furthermore, limited area increases the processing speed, making the extraction very fast.

### 3.2. SUED Segmentation Algorithm

**Figure 6:**
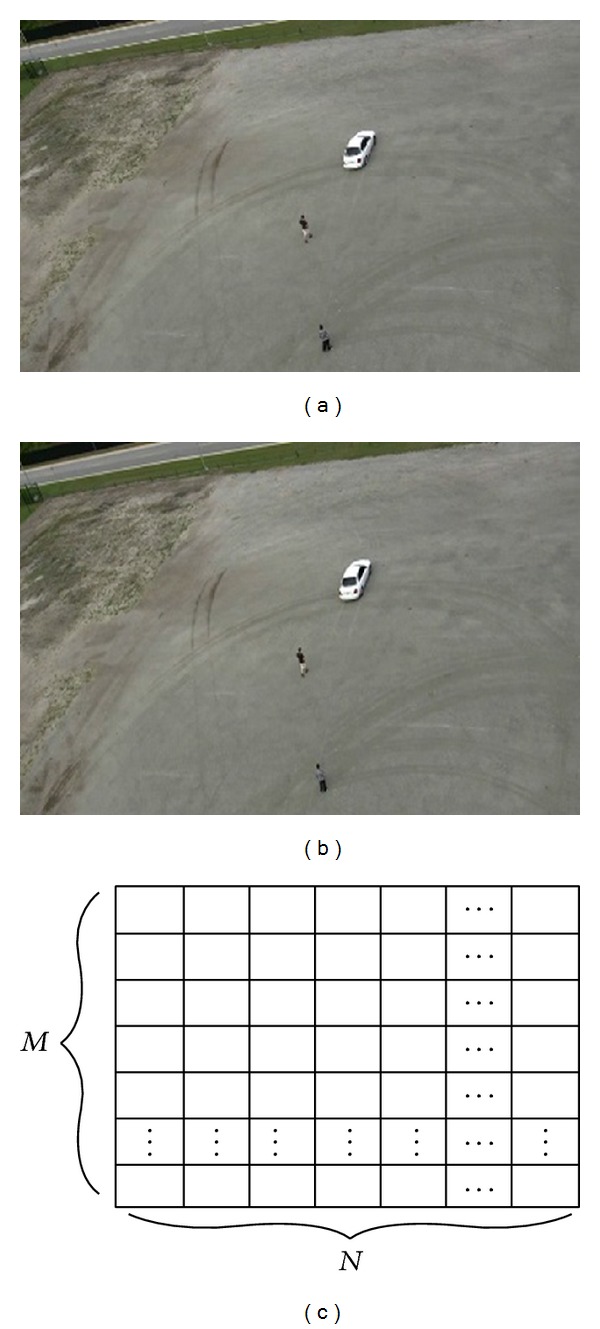
(a) *I*_*B*_(*m*, *n*, *t*) at frame 101. (b) *I*_*B*_(*m*, *n*, *t*) at frame 102. (c) *I*_*B*_(*m*, *n*, *t*) decomposition.

This research used morphological dilation to ensure that extracted moving region contains moving objects. The output of morphological dilation is an edged image. First each frame is decomposed into *M* × *N* uniform and nonoverlapped blocks with the size of *B* × *B* as shown in Figure 6(c). Figures 6(a) and 6(b) show original images at frames 101 and 102 achieved after DMM approach.

**Figure 7:**
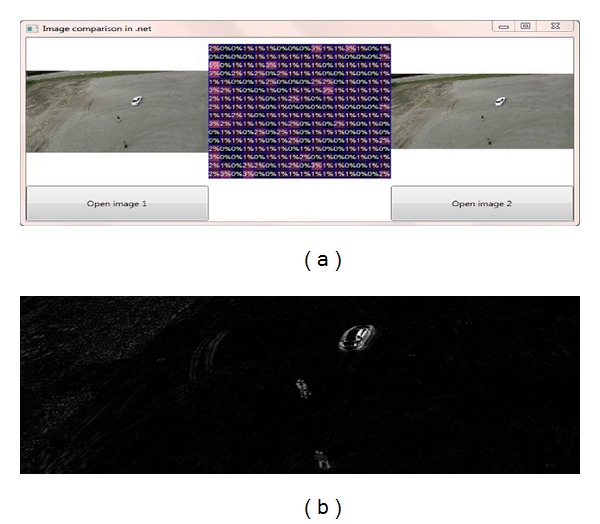
(a) Difference pixel structure of *FD*_*r*_(*m*, *n*, *t*) using (2). (b) Difference image, *FD*_*r*_(*m*, *n*, *t*) using (21).

Let *I*(*x*, *y*, *t*) be the original frame at frame *t* in a video sequence, where (*x*, *y*) denotes a pixel position in the original frame, and let *I*_*B*_(*m*, *n*, *t*) be the corresponding decomposed image, where (*m*, *n*) denotes block position for highest feature density area in the decomposed image which ensures the robustness to noise while the feature makes each block sensitive to motion. *I*_*B*_(*m*, *n*, *t*) is defined by the following equation:

(20) IB(m,n,t)=mean(m,n,t) +αβ2(N1(m,n,t)−N−1(m,n,t))densed  feature,

where (*m*, *n*)  is feature densed block; *α* is the random constant smaller than 1; mean(*m*, *n*, *t*) is mean gray level of all pixels within the block (*m*, *n*) at frame *t*; *N*_1_(*m*, *n*, *t*) is the number of pixels with gray levels greater than mean(*m*, *n*, *t*); *N*_−1_(*m*, *n*, *t*) is the number of pixels with gray levels smaller than mean(*m*, *n*, *t*). From (20), difference image *FD*(*m*, *n*, *t*) of two consecutive block images is obtained by

(21) $$\begin{array}{c} FD_{r}\left(m,n,t\right)=\text{round}\left(\frac{\left|I_{B}\left(m,n,t\right)-I_{B}\left(m,n,t-1\right)\right|}{FD_{\max}\left(t\right)/256}\right), \end{array}$$

where *FD*(*m*, *n*, *t*) is quantized image after rounded operation; *FD*_max⁡_ is maximum value in *FD*(*m*, *n*, *t*). Using (21) the resultant difference is shown in Figures 7(a) and 7(b).

*FD*_*r*_(*m*, *n*, *t*) is filtered by 3 × 3 median filter. *FD*_*f*_(*m*, *n*, *t*) is obtained by following formula:

(22) $$\begin{array}{c} FD_{f}\left(m,n,t\right)=\{\begin{array}{cc} 1, & FD\left(m,n,t\right)\geq T\left(t\right),\\ 0, & \text{otherwise,} \end{array} \end{array}$$

where *T*(*t*) = (Mean of all blocks in *FD*_*r*_(*m*, *n*, *t*) at time *t*)** +** Positive weighting parameter ∗ (Largest peak of histogram of *FD*_*f*_(*m*, *n*, *t*)-Largest peak of histogram of *FD*_*r*_(*m*, *n*, *t*)). Binary image *FD*_*b*_(*m*, *n*, *t*) is obtained by the following condition depicted in Figure 4(a):

(23) $$\begin{array}{c} \text{If}\;\;FD_{f}\left(m,n,t\right)=1,\\ \text{Then }FD_{b}\left(m,n,t\right)⟵\;FD_{f}\left(m,n,t\right),\\ \text{Otherwise},\;FD_{f}\left(m,n,t\right)=0; \end{array}$$

*FD*_*b*_(*m*, *n*, *t*) may have discontinuous boundaries and holes. To ensure that *FD*_*b*_(*m*, *n*, *t*) contains moving object, edge based morphological dilation is proposed here. *FD*_*e*_(*m*, *n*, *t*) represents the edge image Edge(*x*) that can be obtained by gradient operator like Sobel operator. *FD*_*d*_(*m*, *n*, *t*) is the edge based morphological dilation of the image. *FD*_*e*_(*m*, *n*, *t*) is obtained by the following formula:

(24) FDd(m,n,t) =FDe(m,n,t)∪{x ∣ x=i+j;i∈FDe(m,n,t)};j∈L;Edge(x)≠0,

where *L* is the structuring element that contains elements *i*, *j*.

**Figure 8:**
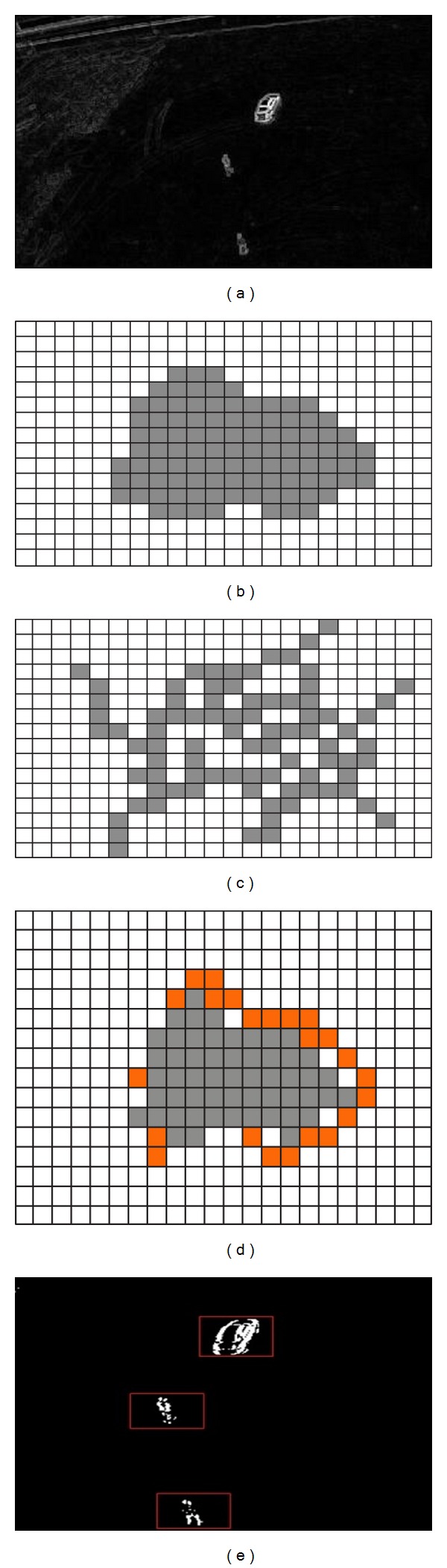
(a) *FD*_*b*_(*m*, *n*, *t*). (b) Conventional dilation: *FD*_*b*_(*m*, *n*, *t*) ⊕ *L* = {*x* | *x* = *i* + *j*; *i* ∈ *FD*_*e*_(*m*, *n*, *t*); *j* ∈ *L*}. (c) Analysis of edge based dilation from *FD*_*e*_(*m*, *n*, *t*). (d) Edge based dilation: *FD*_*d*_(*m*, *n*, *t*) = *FD*_*e*_(*m*, *n*, *t*) ∪ {*x* | *x* = *i* + *j*; *i* ∈ *FD*_*e*_(*m*, *n*, *t*)}; *j* ∈ *L*; Edge(*x*) ≠ 0. (e) Output after SUED: *FD*_*e*_(*m*, *n*, *t*).

Equation (24) is considered as edge based dilation that is expected to avoid undesired regions to be integrated in the result according to edge based morphological dilation characteristics as shown in Figure 8(d). Here {*x* | *x* = *i* + *j*; *i* ∈ *FD*_*e*_(*m*, *n*, *t*)} is the conventional dilation after obtaining *FD*_*e*_(*m*, *n*, *t*) which can be defined from *FD*_*b*_(*m*, *n*, *t*) as *FD*_*b*_(*m*, *n*, *t*) ⊕ *L* = {*x* | *x* = *i* + *j*; *i* ∈ *FD*_*b*_(*m*, *n*, *t*); *j* ∈ *L*} shown in Figure 8(b).

This research proposed the following segmentation approach: segmentation using edged based dilation (SUED) algorithm for moving object extraction from UAV aerial images after extracting frame from DMM approach.

1. Start
2. *FD*_*r*_(*m*, *n*, *t*) ← Decomposed image between 2 frames,  *I*_*B*_(*m*, *n*, *t*) − *I*_*B*_(*m*, *n*, *t* − 1) at *t* and *t* − 1  time.
3. *FD*_*f*_(*m*, *n*, *t*) ← *FD*_*r*_(*m*, *n*, *t*)
4. *FD*_*b*_(*m*, *n*, *t*) ← *FD*_*f*_(*m*, *n*, *t*)
5. *FD*_*e*_(*m*, *n*, *t*) ← *FD*_*b*_(*m*, *n*, *t*)
6. *FD*_*d*_(*m*, *n*, *t*) ← *FD*_*e*_(*m*, *n*, *t*)
7. End.

## 4. Experiment and Discussion

For the experiment purpose this research used IMAGE PROCESSING LAB (IPLAB) available at http://www.aforge.net/ which is embedded with Visual Studio 2012 using C sharp programming language. Proposed experimental analysis evaluates dynamic motion modeling (DMM) first and then experimented the proposed SUED embedded with DMM to extract moving object from UAV aerial images.

Let *FD*_*e*_(*m*, *n*, *t*) be the labeled result of the SUED segmentation algorithm shown in Figure 8(e). Each region in this image indicates coherence in intensity and motion. Moving objects are discriminated from backgrounds by the fusion module that combines DMM (dynamic motion module) and SUED (segmentation using edge based dilation). Let SUED(*i*, *t*) be a SUED region in *FD*(*m*, *n*, *t*) and *A*_*s*_(*i*, *t*) the corresponding area. Let *A*_*c*_(*i*, *t*) be the area of union of the SUED(*i*, *t*), and the coverage ratio *P*(*i*, *t*) is defined as

(25) $$\begin{array}{c} P\left(i,t\right)=\frac{A_{c}\left(i,t\right)}{A_{s}\left(i,t\right)}. \end{array}$$

If the value of *P*(*i*, *t*) is greater than a given fusion threshold *T*_*s*_, then SUED(*i*, *t*) is considered as foreground, otherwise background. In general, threshold *T*_*s*_ varies for different sequence. In this research, threshold *T*_*s*_ is always set to 0.99. It is very close to 1 and does not need to be adjusted as obtained area can contain complete desired object. There may be some smaller blobs in the resulting SUED(*i*, *t*). Those regions with the areas smaller than the threshold *T*_*s*_ are considered as noisy regions. Figure 8(e) shows the extracted moving object as the resultant of fusion for DMM and SUED methodology.

### 4.1. Datasets

This research used two UAV video datasets (actions1.mpg and actions2.mpg) from Center for Research in Computer Vision (CRCV) in University of Central Florida (http://crcv.ucf.edu/data/UCF_Aerial_Action.php). These video datasets were obtained using R/C controlled blimp equipped with HD camera. The collection represents a diverse pool of action features at different heights and aerial view points. Multiple instance of each action was recorded at different flying altitudes which ranged from 400 to 500 feet and was performed with different actors.

### 4.2. Results

This research extracted 395 frames using 1 frame/second video frame rate from actions1.mpg video datasets and 529 frames using same frame rate from actions2.mpg video datasets. Frame size is 355 × 216. Figures 6(a) and 6(b) show two consecutive frames from (101th and 102th frames) after DMM step from actions1.mpg. Figures 7(a) and 7(b) show pixel structures of *FD*_*r*_(*m*, *n*, *t*); Figure 8(a) shows *FD*_*b*_(*m*, *n*, *t*); Figure 8(b) shows conventional dilation of *FD*_*b*_(*m*, *n*, *t*), while Figures 8(c) and 8(d) show edge based dilation. Finally Figure 8(e) shows resultant of the proposed DMM and SUED based moving object detection.

#### 4.2.1. DMM Evaluation

Figures 1(a), 1(b), and 1(c) show three search windows which are depicted as the black rectangle on selected frame in *t* time, *FD*(*m*, *n*). The brighter the moments value for pixels, the higher the probability that search windows belong to the moving object. In Table 2 measurement of moments is shown.

**Figure 9:**
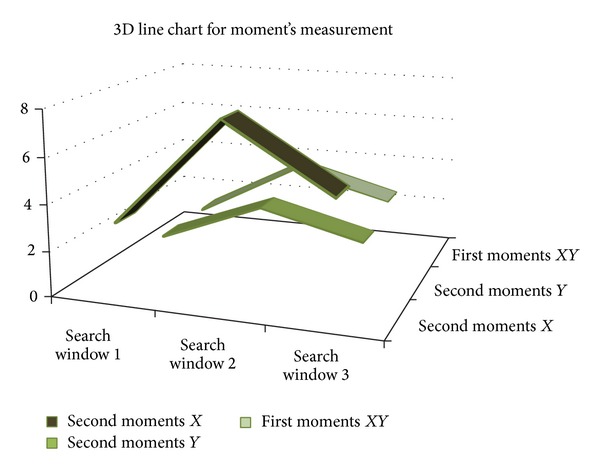
3D line chart for moment's measurement.

In Table 2, search window 2 shows the highest moment quantity among 3 search windows which indicates that search window 2 contains the highest probability to contain moving objects.  Figure 9 shows 3D line chart to depict the highest probability of pixels intensity in search window 2. This research used search window 2 as based on the probability of the highest moments distribution in the search windows to extract moving object using SUED, because it limits the scope of segmentation within smaller area, reduces complexity, and reduces time. This means that the probability of the extraction is getting lost because a similar coloured object in the background is reduced. Furthermore limited area increases the processing speed by making the extraction faster.

#### 4.2.2. SUED Evaluation

This section presents some of the experimental analysis and results for the proposed SUED algorithm. The evaluation of the proposed approach was tested on actions1.mpg and actions2.mpg video analysis. In order to evaluate SUED algorithm, two metrics, detection rate (DR) and the false alarm rate (FAR), are defined. These metrics are based on the following parameters.

- True positive (TP): detected regions that correspond to moving object.
- False positive (FP): detected regions that do not correspond to a moving object.
- False negative (FN): moving object not detected.
- Detection rate or precision rate, DR = (TP/(TP + FN)) × 100%.
- False alarm rate or recall rate, FAR = (FP/(TP + FP)) × 100%.

From dataset actions1.mpg, this research extracts 395 frames using 1 frame per second, and from actions2.mpg, this research extracted 529 frames using the same frame rate. Details of measurement for true positive (TP), false positive (FP), false negative (FN), detection rate (DR), and false alarm rate (FAR) are mentioned in Table 3.

**Figure 10:**
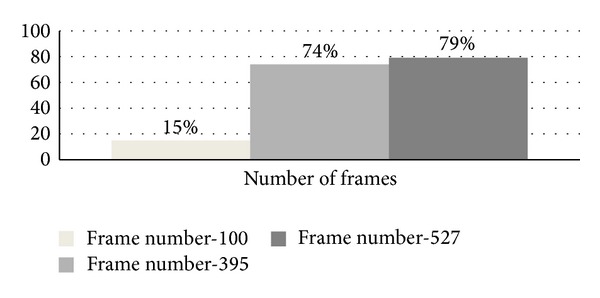
Detection rate or precision rate.

Detection rate increases if the number of input frames is increased. The detection rate of the given total frame for two video datasets is displayed in Figure 10.

**Figure 11:**
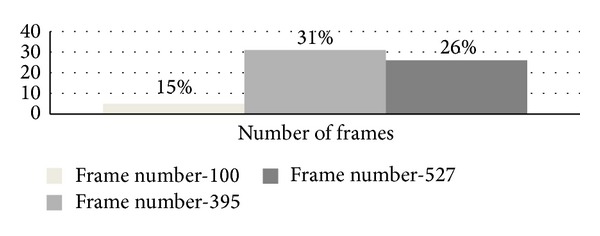
False alarm rate or recall rate.

The false alarm rate for the given number of frame from two video datasets is given in Figure 11.

**Figure 12:**
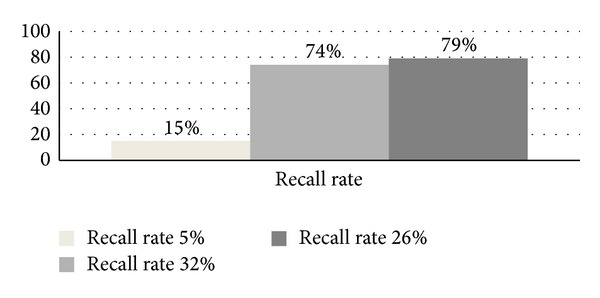
RPC characterization.

Recall and precision rate (RPC) characterizing the performance of the proposed research are given in Figure 12.

This research measures detection and false alarm rate based on the number of frames extracted from each video dataset input. Compared with [1, 15, 36, 40], this research used frame difference approach where moments feature for motion modeling was not included. This research achieved detection rate of 79% (for video datasets actions2.mpg) which is a good indication to handle motion of moving object in future research. Thus, the proposed DMM model is helpful to reduce segmentation task by providing the highest probability area to be segmented using the proposed SUED instead of segmenting the whole area for the given frame by increasing processing speed.

## 5. Conclusion

The primary purpose of this research is to apply moments based dynamic motion model under the proposed frame difference based segmentation approach which ensures that robust handling of motion as translation invariant, scale invariant, and rotation invariant moments value is unique. As computer vision leverages probability theory, this research used moments based motion analysis which provides search windows around the original object and limits the scope of SEUD segmentation to a smaller area. This means that the probability of extraction is getting lost because a similar colored object in the background is reduced. Since moments are the unique distribution of pixel intensity, so experimental result of the proposed DMM and SUED is very promising for robust extraction of moving object from UAV aerial images. Judging from the previous research in computer vision field, it is certain that the proposed research will facilitate UAV operator or related researchers for further research or investigation in areas where access is restricted or rescue areas, human or vehicle identification in specific areas, crowd flux statistics, anomaly detection and intelligent traffic management, and so forth.

## Figures and Tables

**Table 1:** Comparison of parameters for various motion analysis approaches.

Parameters	Approaches			
	Illumination compensation	Parallel filtering	Contextual information	Long term motion analysis
Strong parallax situation	No	No	No	Yes
Level of motion detection	Low	Low	Low	Low and high
Environmental condition	No	No	Yes	Yes
Lot of parameters	Yes	Yes	Yes	No
Computational complexity	High	High	High	High

**Table 2:** Moments measurement from different search windows for SUED.

Search window	Zeroth moment	First moment *XY*	Second moment *X*	Second moment *Y*
1	9.8*E*6	1.21*E*11	3.1*E*11	1.16*E*11
**2**	**1.6** ***E*** **7**	**3.94** ***E*** **11**	**8.69** ***E*** **11**	**2.95** ***E*** **11**
3	9.3*E*6	2.49*E*11	5.03*E*11	1.84*E*11

**Table 3:** Details of measurement of true positive (TP), false positive (FP), false negative (FN), detection rate (DR), and false alarm rate (FAR).

Datasets	Number of frames	True positive (TP)	False positive (FP)	False negative (FN)	Detection rate (DR)	False alarm rate (FAR)
Actions1.mpg	395	200	100	75	75%	31%
Actions2.mpg	529	320	113	83	79%	26%
